# Supplementary material for: Development of a resilience assessment tool for cardiac care pathways in Europe: a mixed-methods study
Source: BMJ Open. 2026 Feb 6;16(2):e110266. doi: 10.1136/bmjopen-2025-110266 (PMC12887496; doi:10.1136/bmjopen-2025-110266)

## Supplementary file 3 - RESIL-Card survey


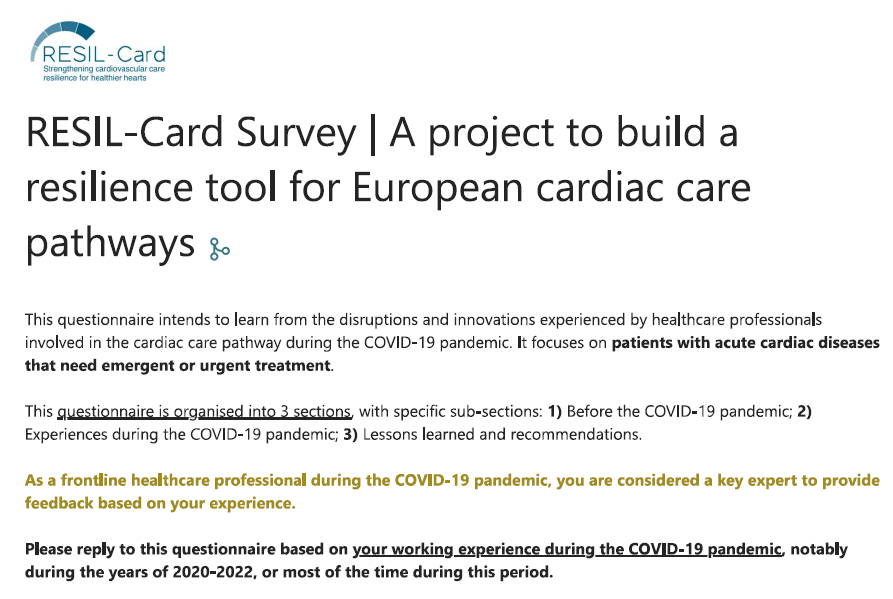


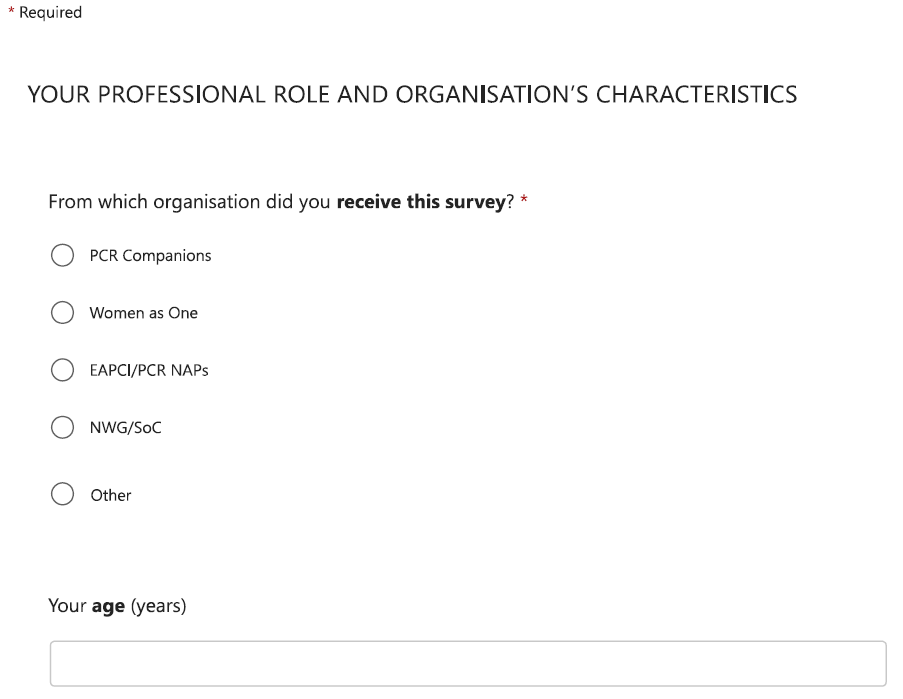


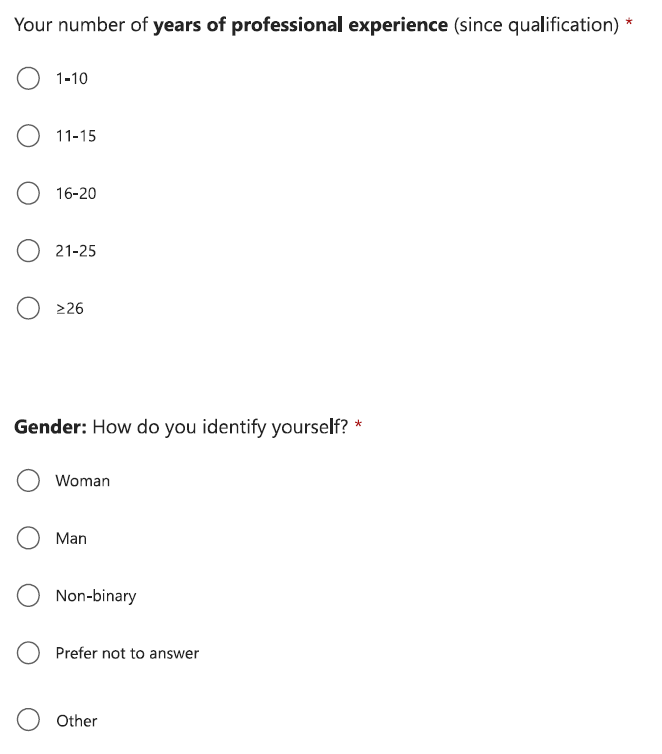


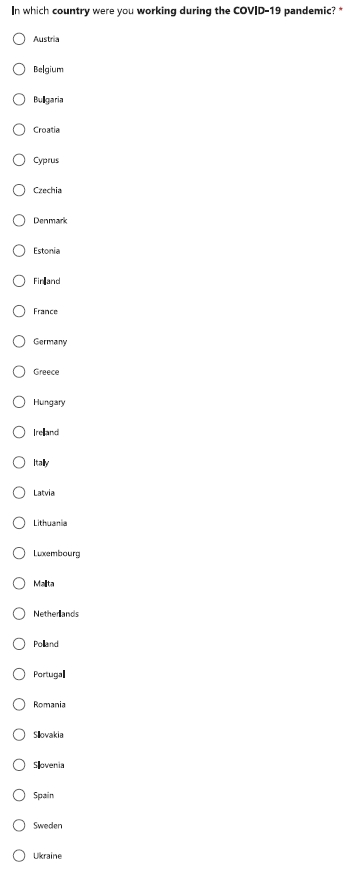


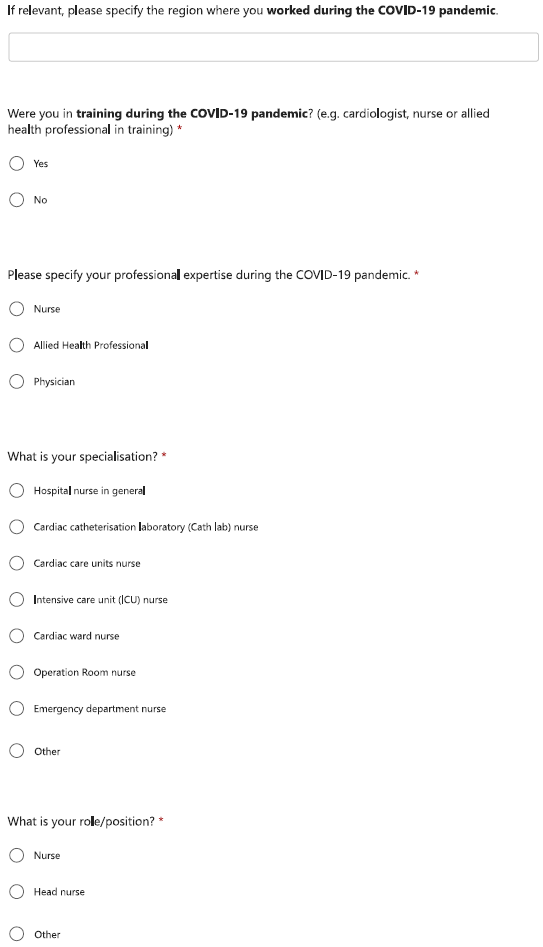


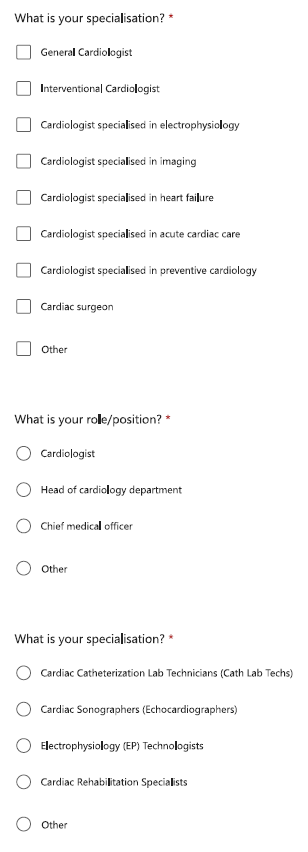


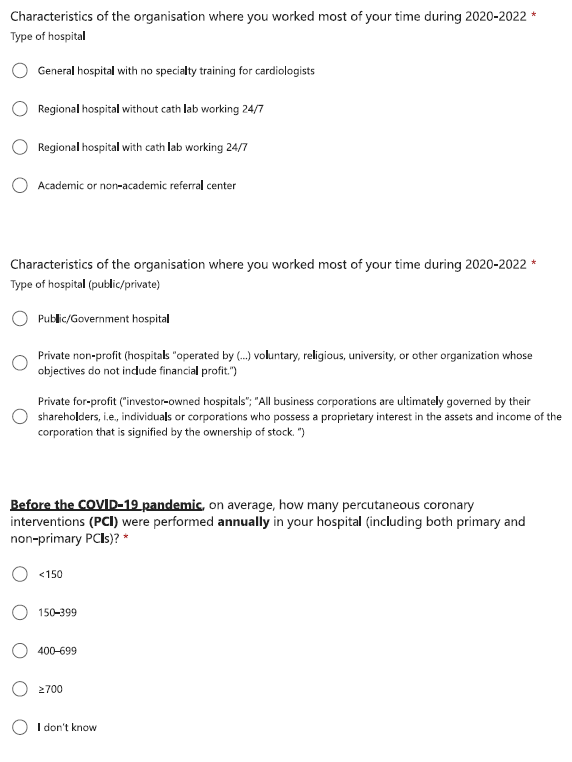


### Section 1 - before the COVID-19 pandemic


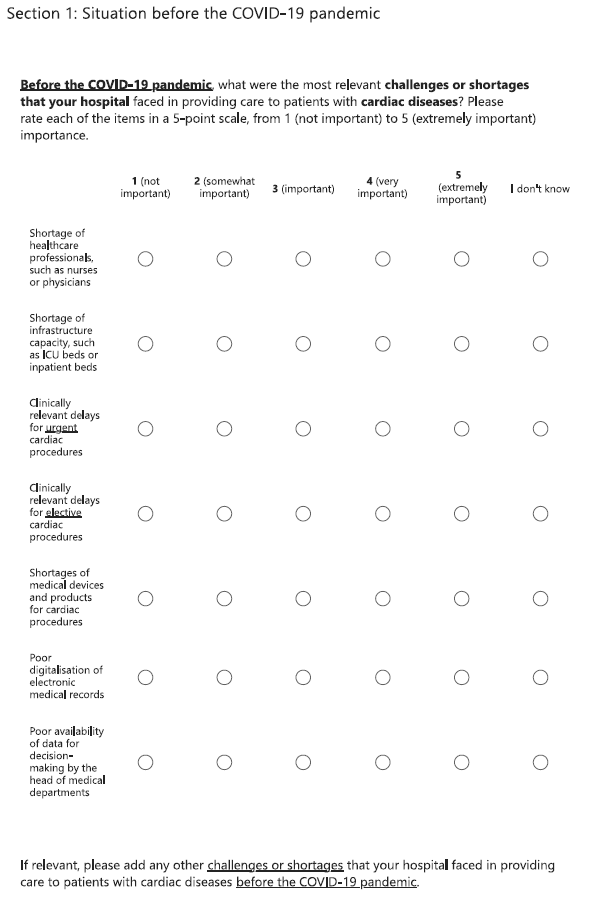


### Section 2 - during the COVID-19 pandemic


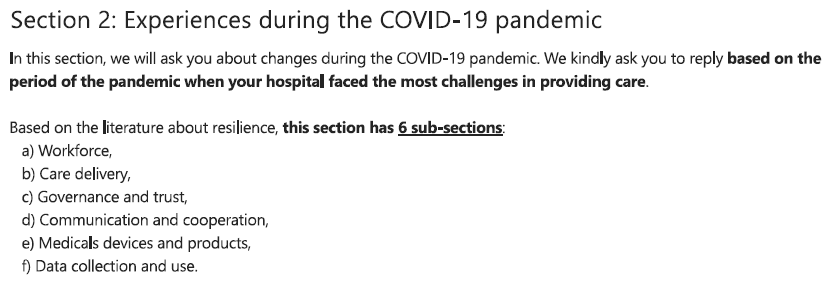


### a) Workforce


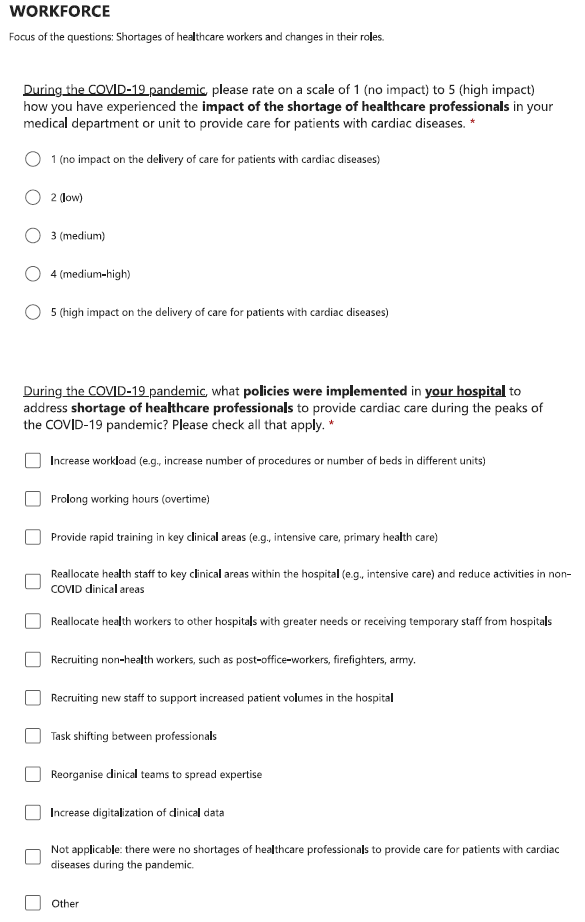


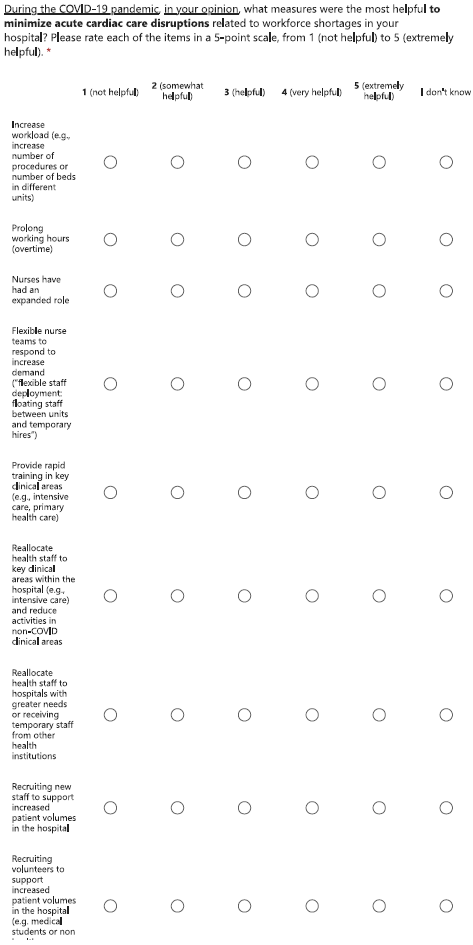


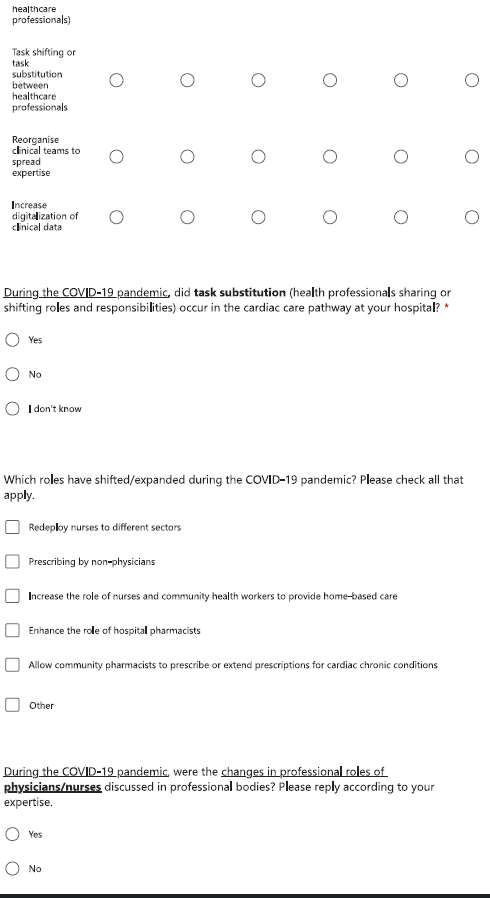


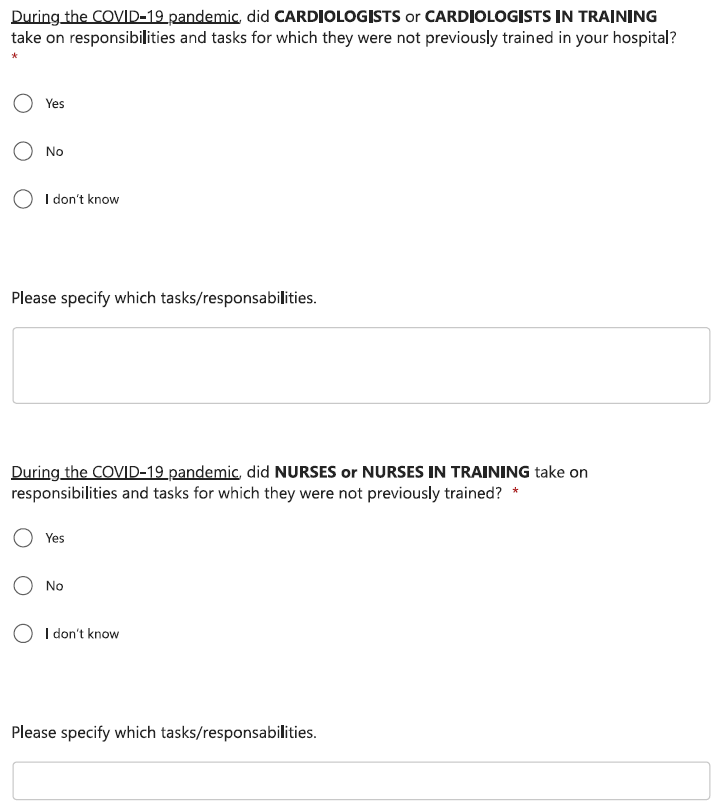


### b) Care delivery


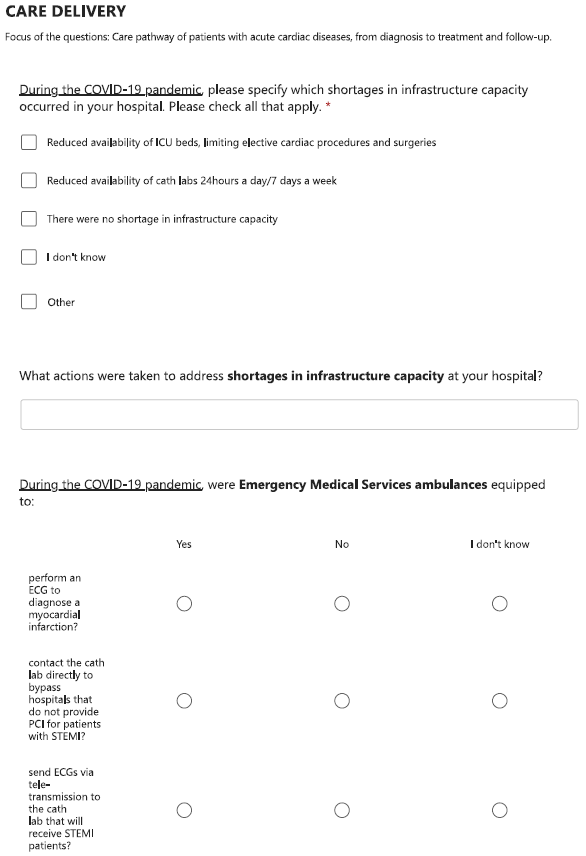


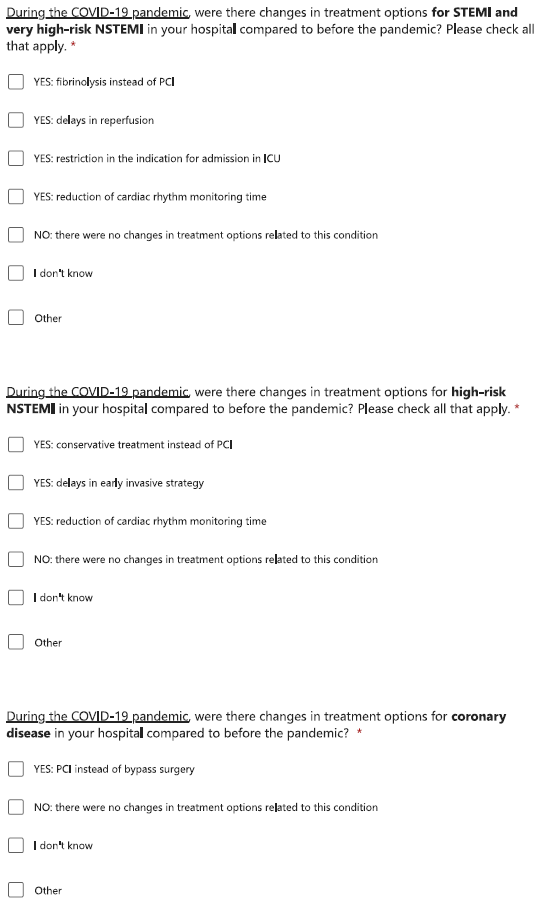


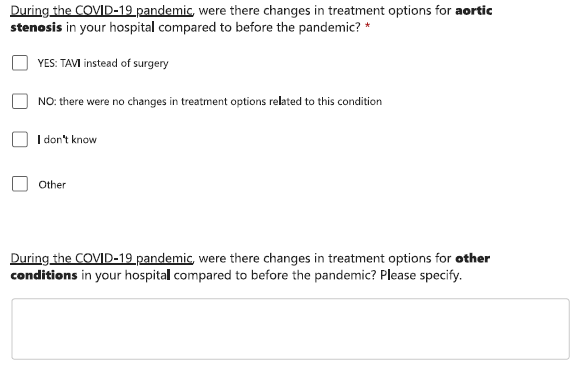


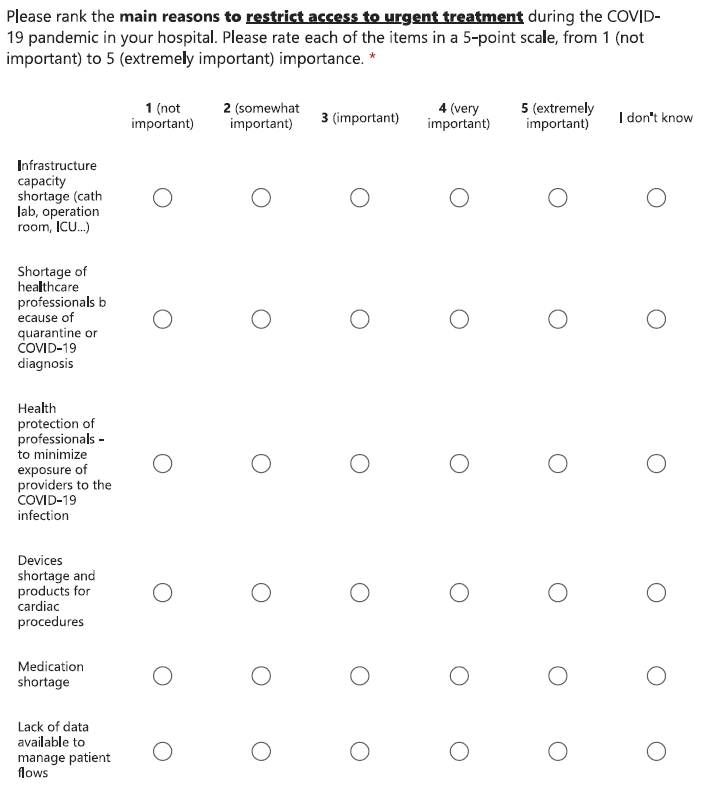


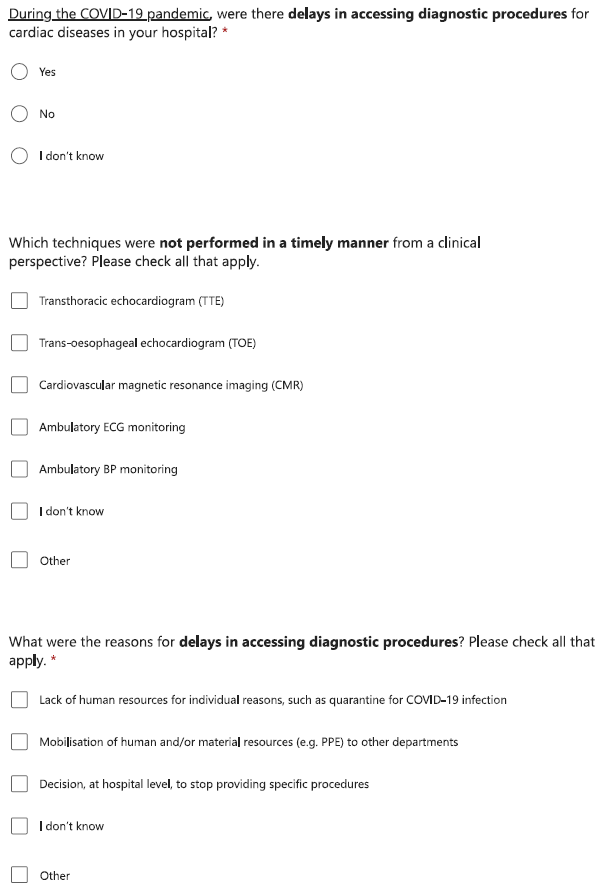


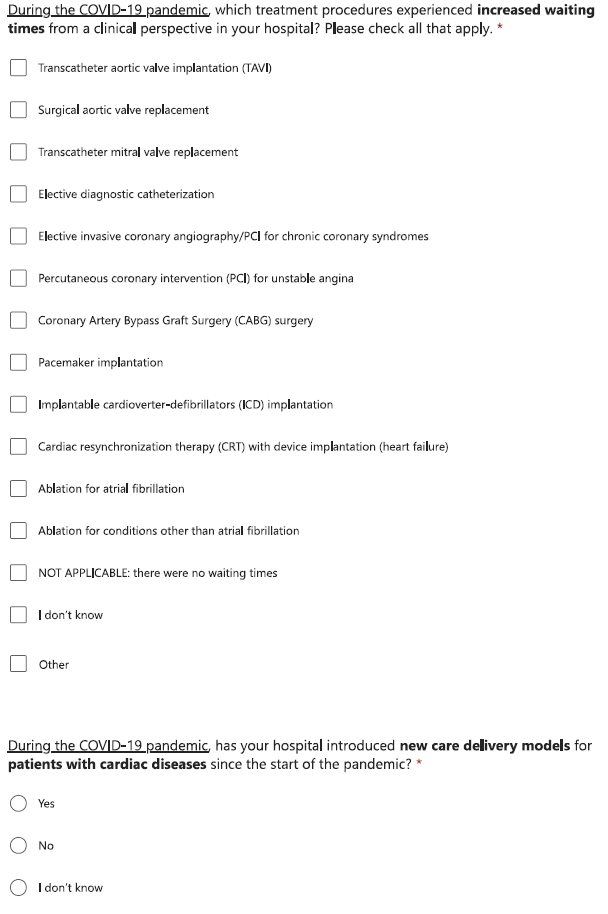


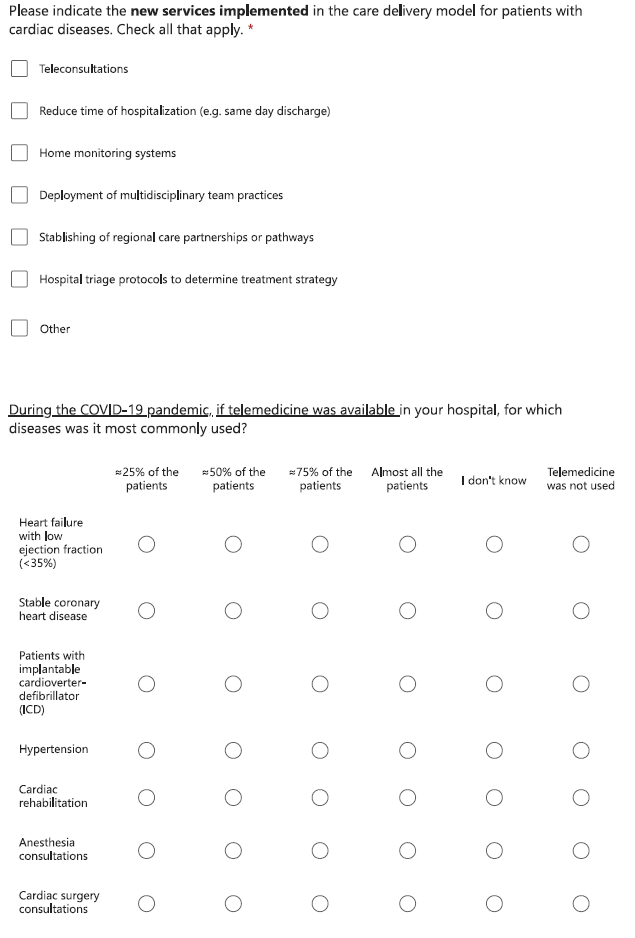


### c) Governance and trust


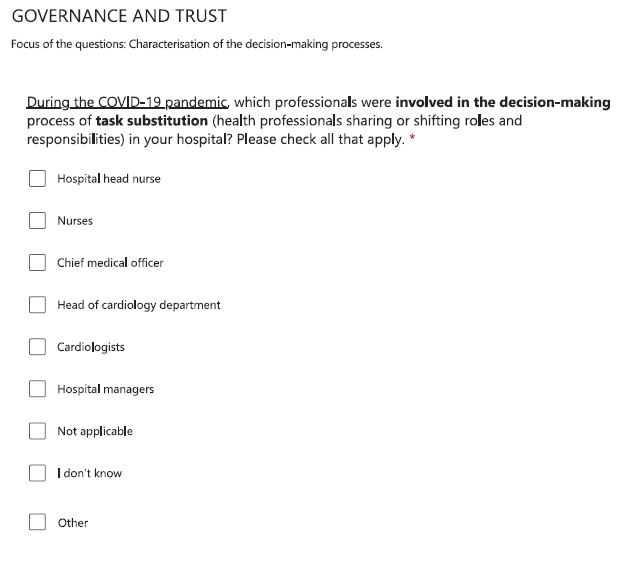


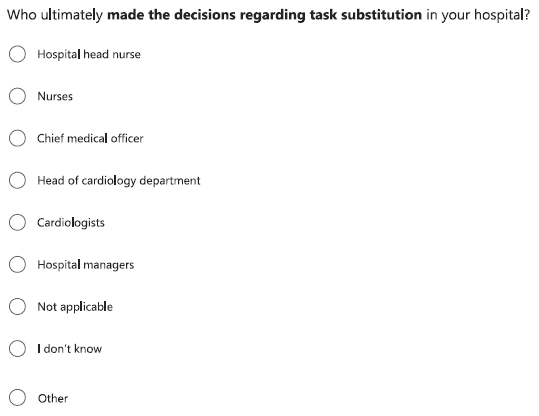


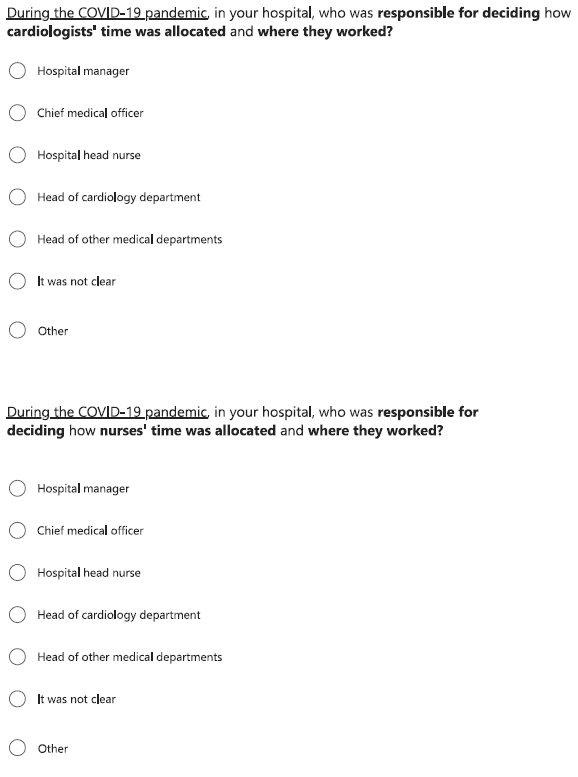


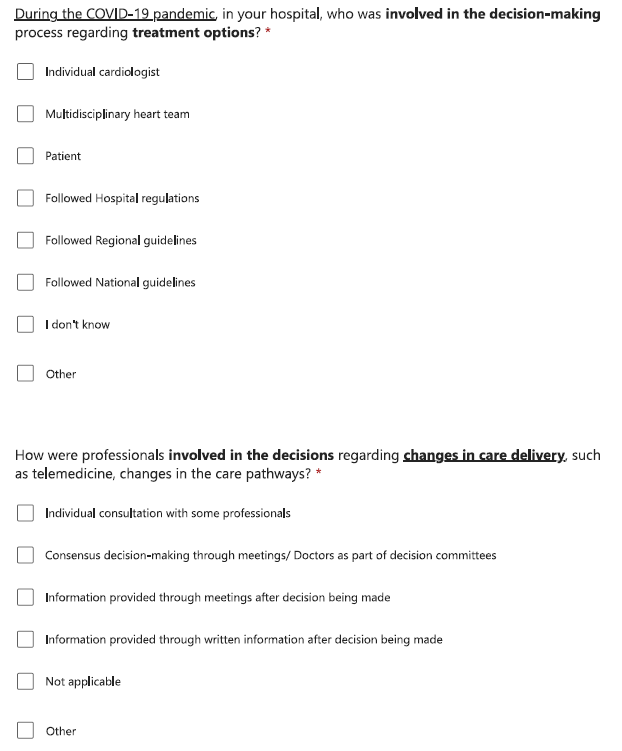


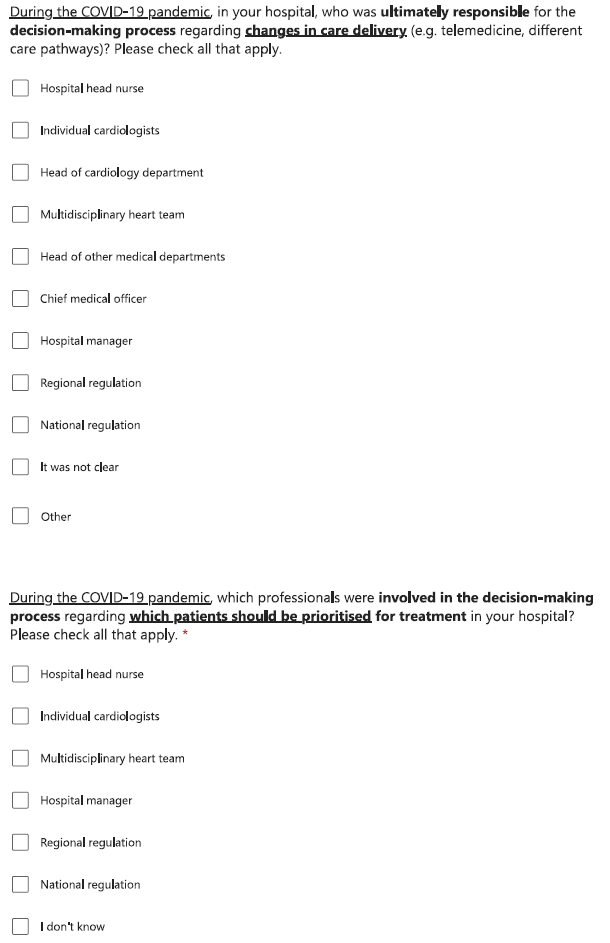


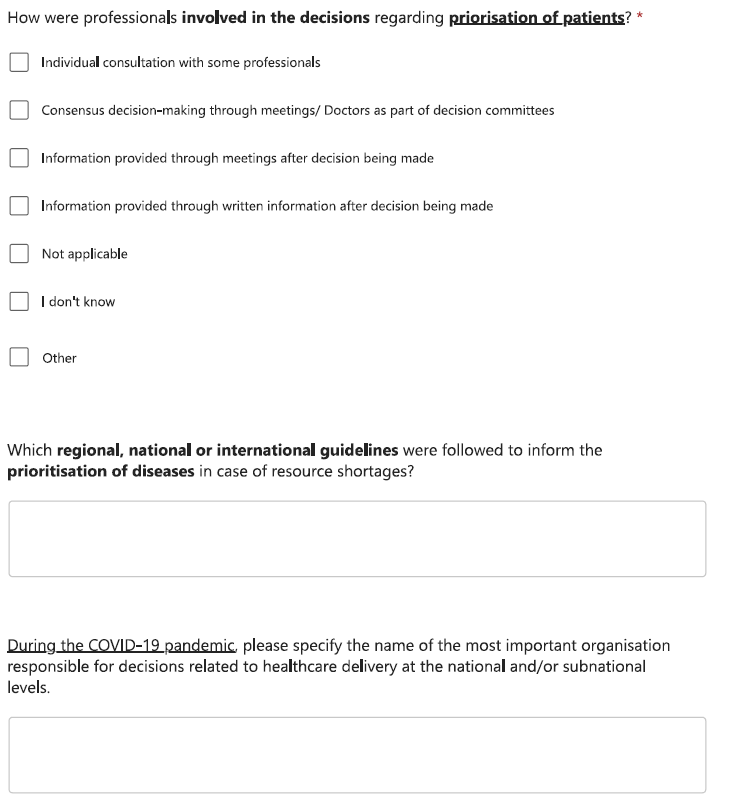


### d) Communication and cooperation


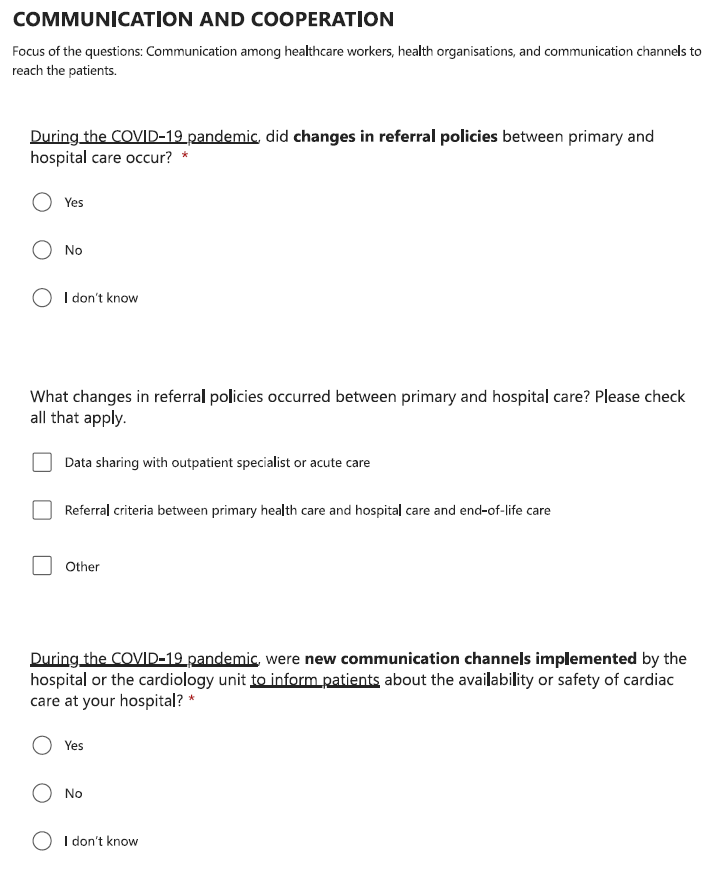


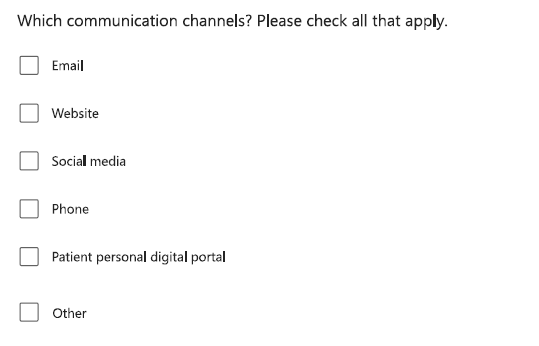


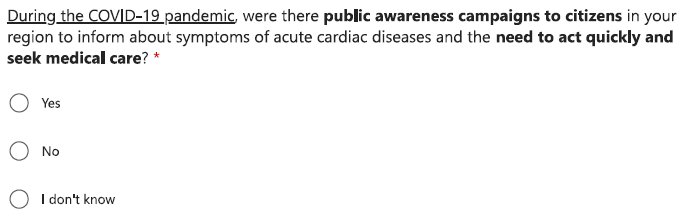


### e) Medicals devices and products


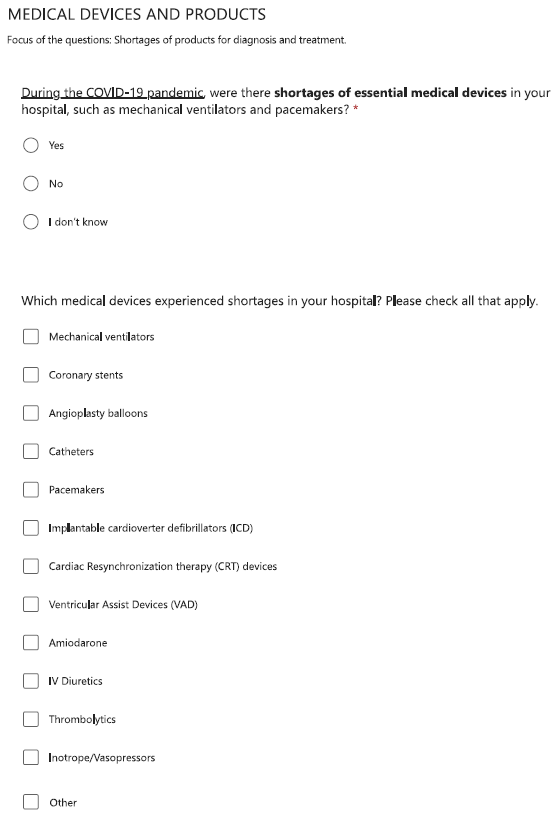


### f) Data collection and use


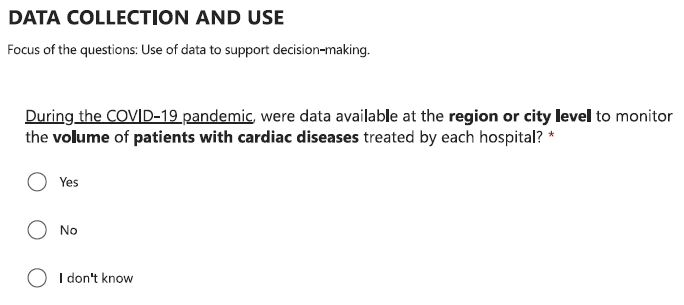


### Section 3 – after the COVID-19 pandemic


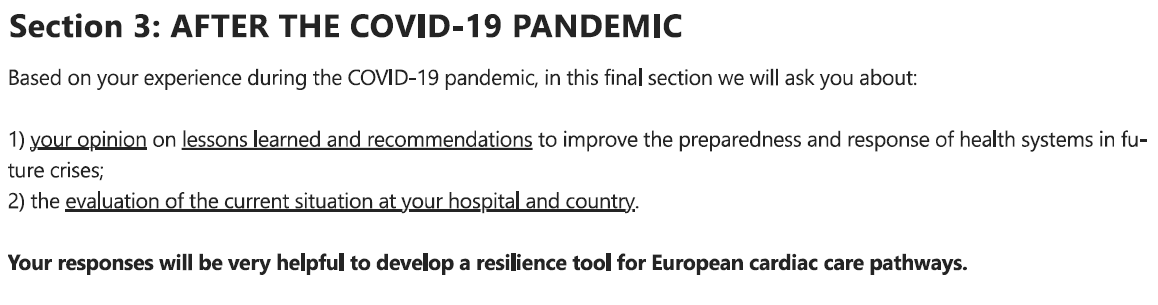


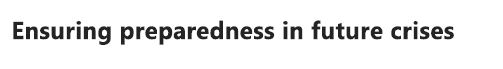


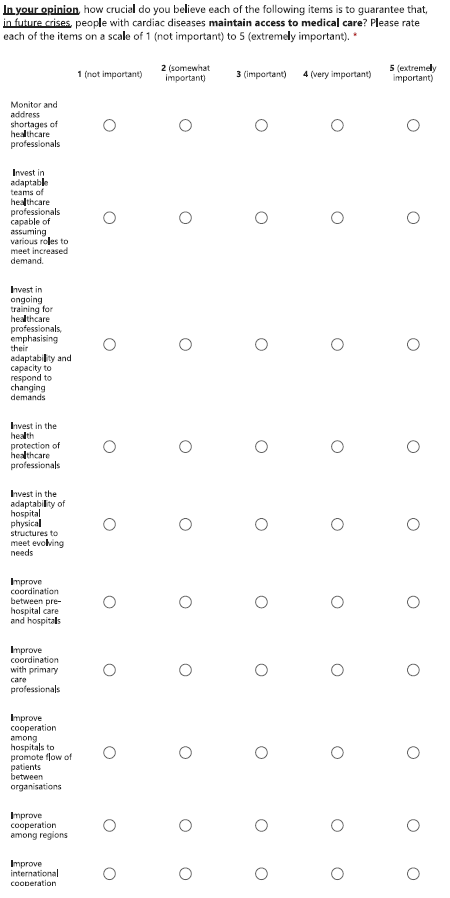


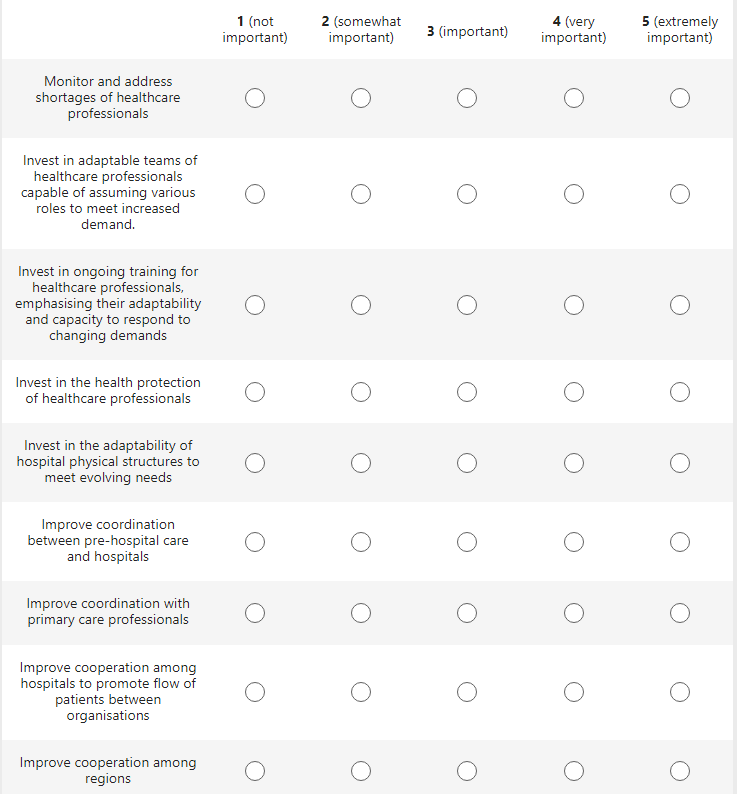


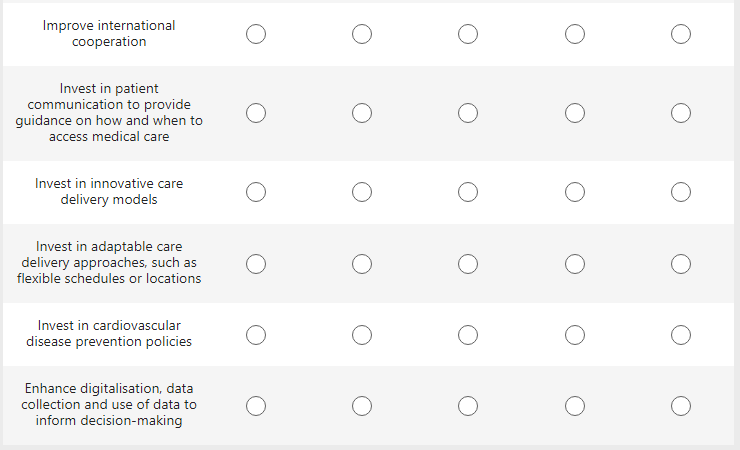


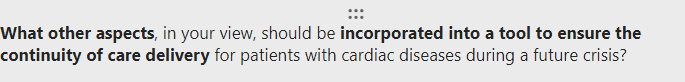


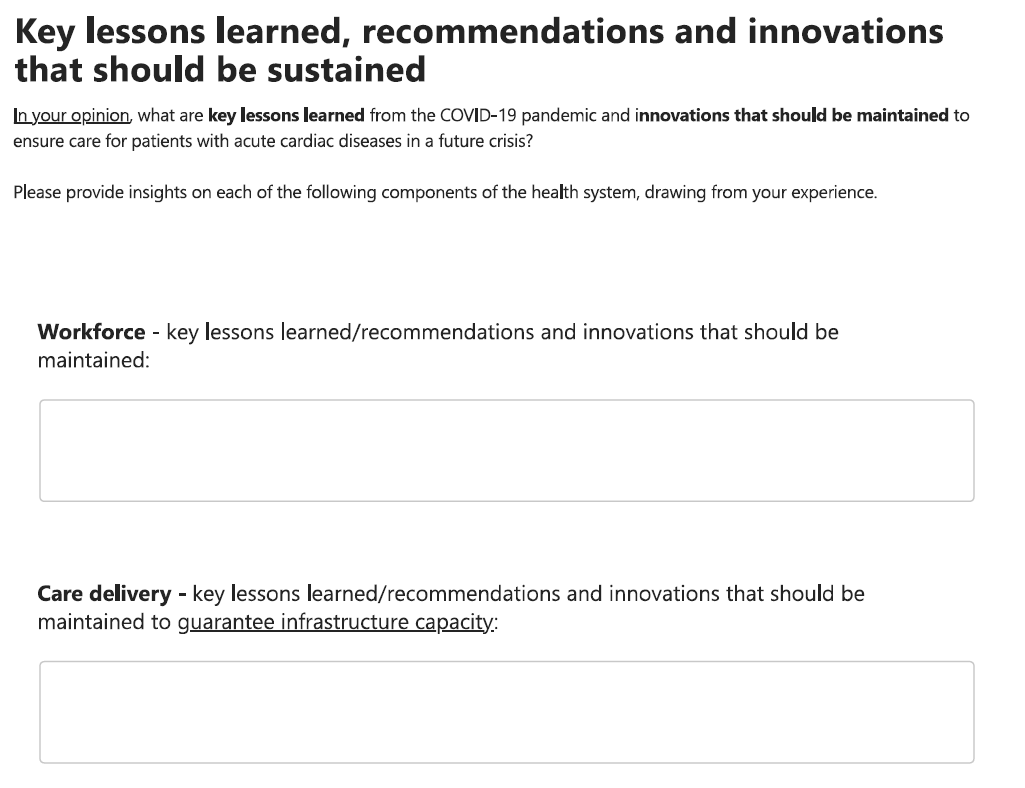


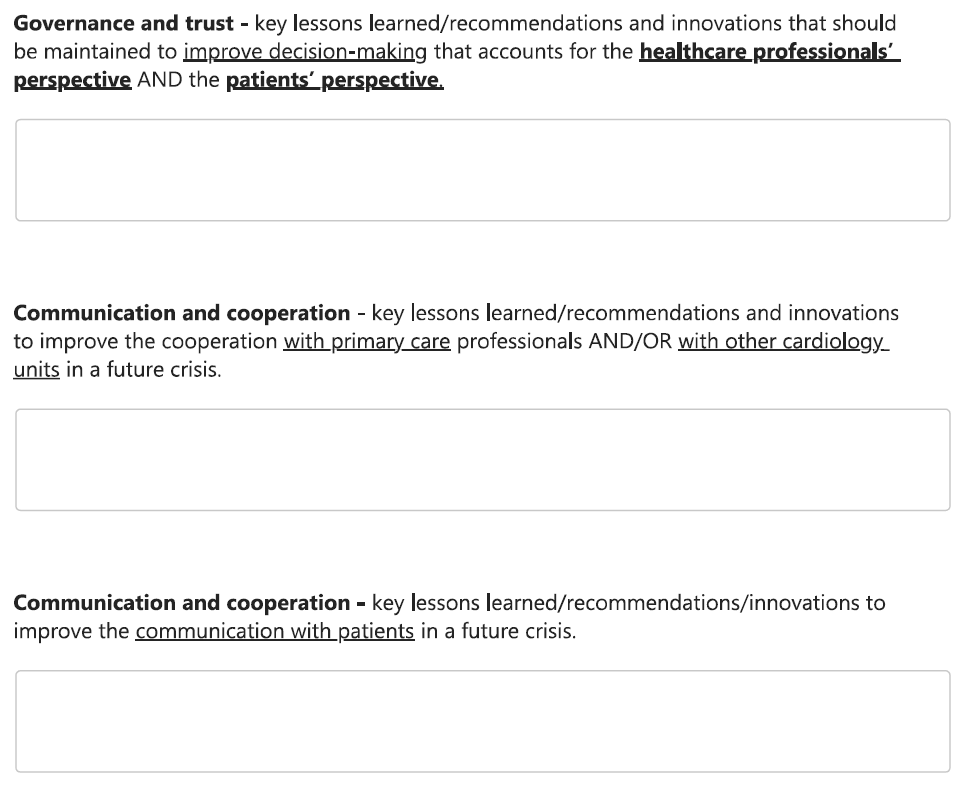


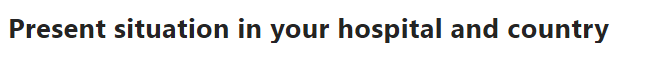


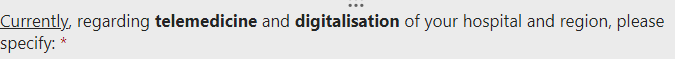


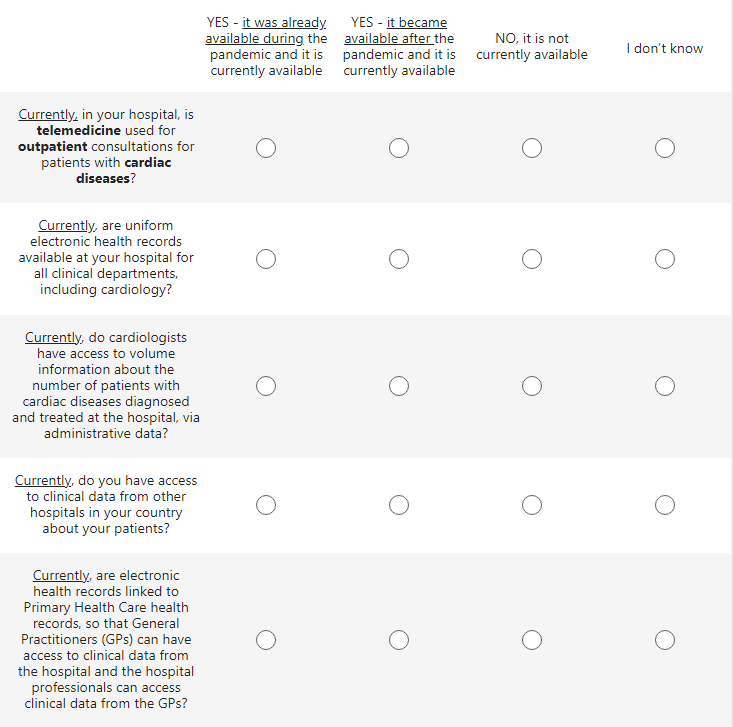


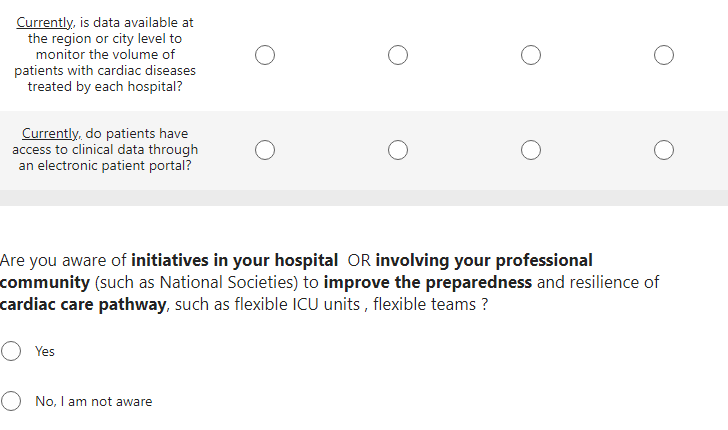


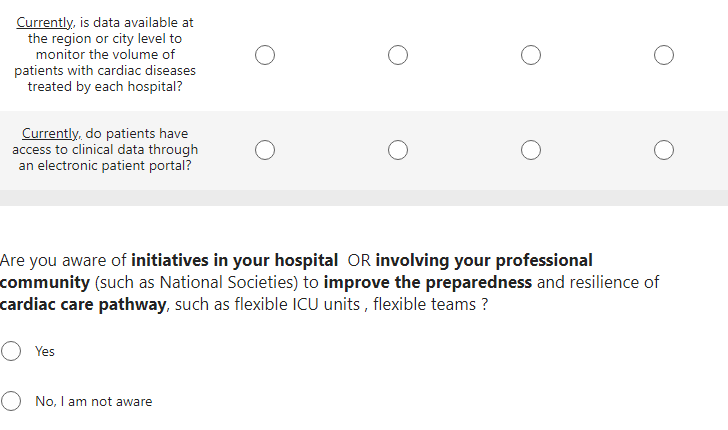


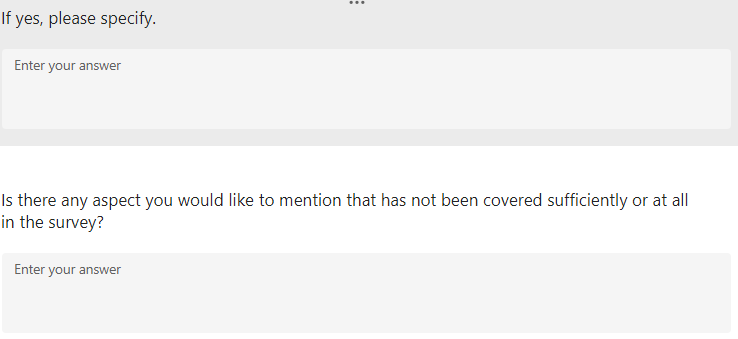


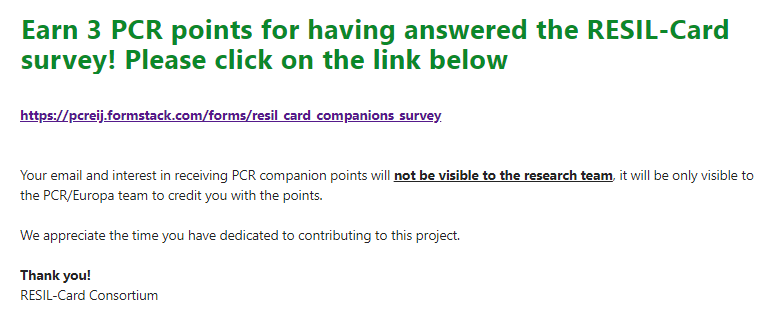

Supplement: online supplemental file 3 [file bmjopen-16-2-s003.docx]
