# Supplementary material for: Development of a resilience assessment tool for cardiac care pathways in Europe: a mixed-methods study
Source: BMJ Open. 2026 Feb 6;16(2):e110266. doi: 10.1136/bmjopen-2025-110266 (PMC12887496; doi:10.1136/bmjopen-2025-110266)

## Supplemental file 8 - Survey results, Section1: Challenges before the COVID-19 pandemic

66 respondents (37%) rated shortage of infrastructure capacity as a very important and extremely important challenge to care delivery before the pandemic and 64 (36%) rated nurses or physicians shortages as a relevant challenge.


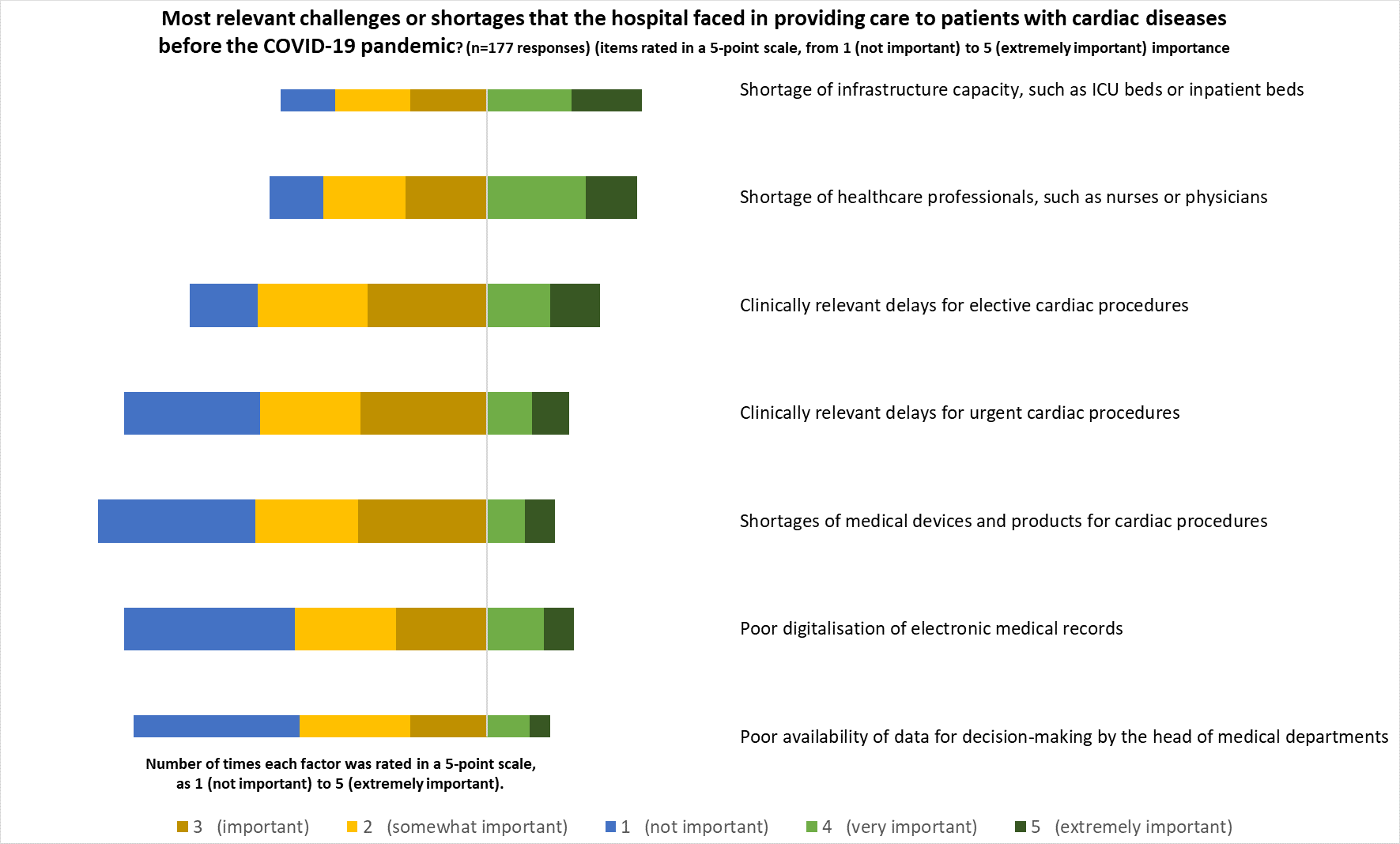

Supplement: online supplemental file 8 [file bmjopen-16-2-s008.docx]
