# Supplementary figures and images for: Development of a resilience assessment tool for cardiac care pathways in Europe: a mixed-methods study
Source: BMJ Open. 2026 Feb 6;16(2):e110266. doi: 10.1136/bmjopen-2025-110266 (PMC12887496; doi:10.1136/bmjopen-2025-110266)

## Supplemental file 10 - Survey results, Section3: Lessons learned after the COVID-19 pandemic


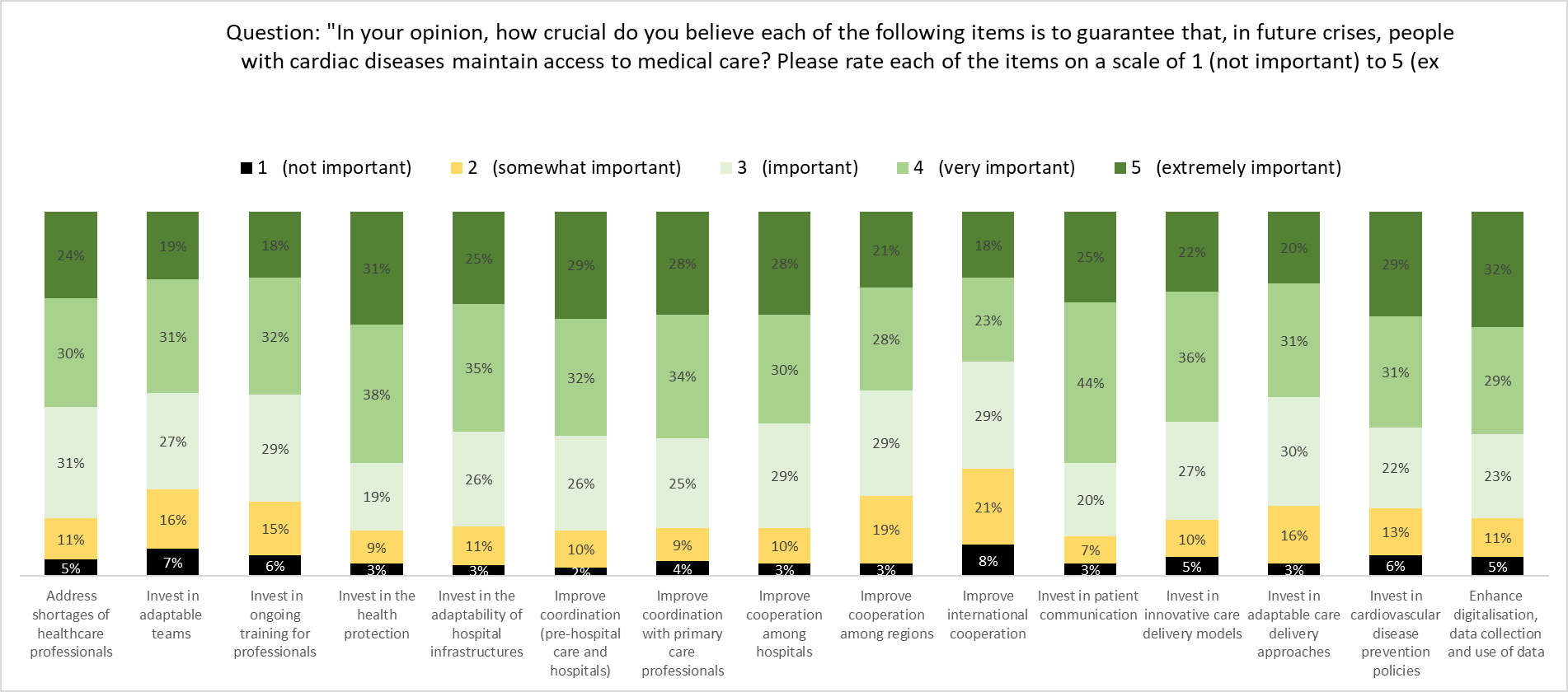

Supplement: online supplemental file 10 [file bmjopen-16-2-s010.docx]
