# Supplementary material for: Development of a resilience assessment tool for cardiac care pathways in Europe: a mixed-methods study
Source: BMJ Open. 2026 Feb 6;16(2):e110266. doi: 10.1136/bmjopen-2025-110266 (PMC12887496; doi:10.1136/bmjopen-2025-110266)

## Supplementary file 11 - Themes identified to develop the resilience tool, per resilience dimension

Survey themes were complemented and refined with the focus groups discussions, per resilience dimension, to develop the resilience tool


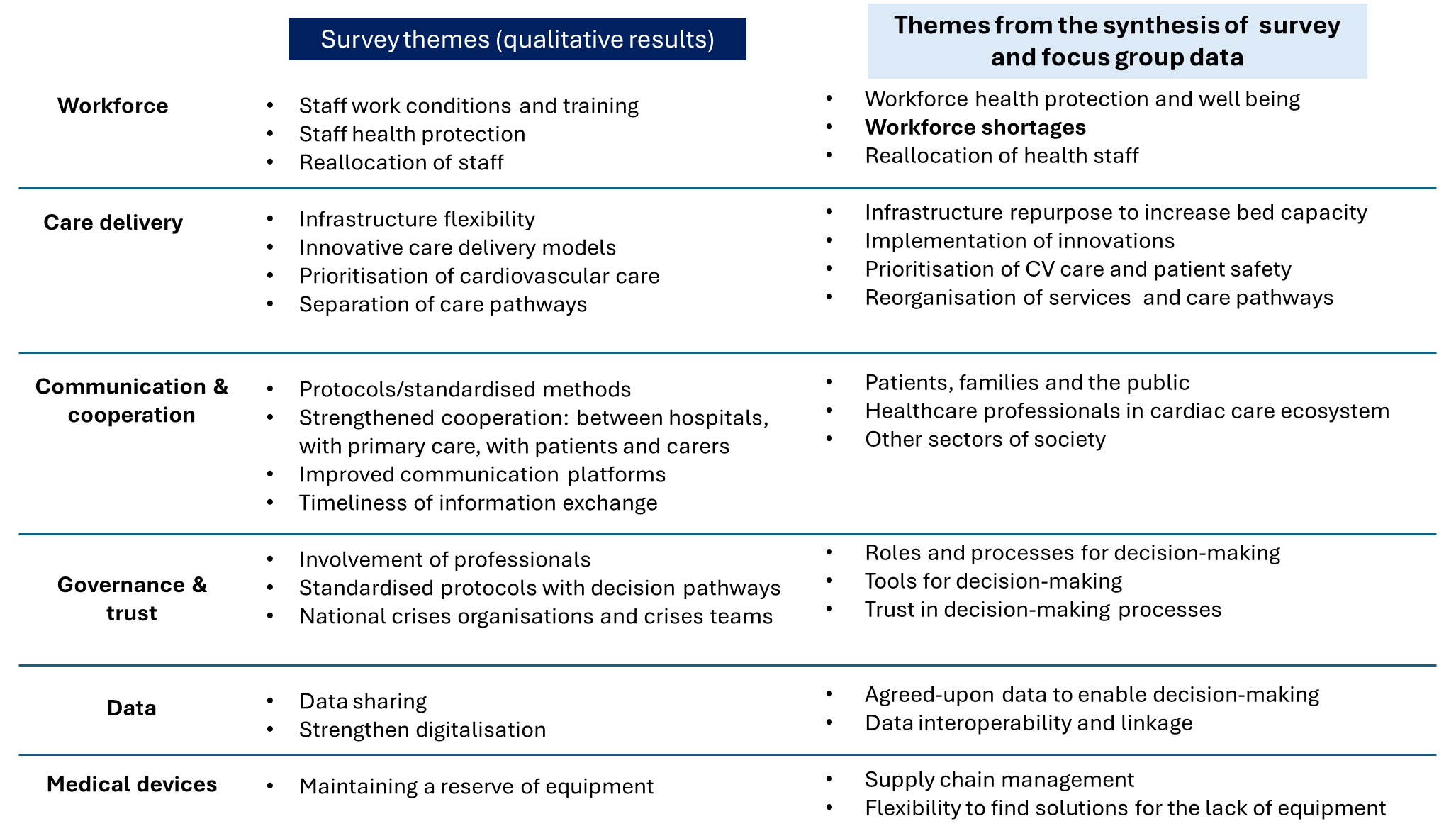

Supplement: online supplemental file 11 [file bmjopen-16-2-s011.docx]
