# Supplementary material for: Development of a resilience assessment tool for cardiac care pathways in Europe: a mixed-methods study
Source: BMJ Open. 2026 Feb 6;16(2):e110266. doi: 10.1136/bmjopen-2025-110266 (PMC12887496; doi:10.1136/bmjopen-2025-110266)
Supplement: Supplementary file 12 [file bmjopen-2025-110266.reviewer_comments.pdf]

PEER REVIEW HISTORY

BMJ Open publishes all reviews undertaken for accepted manuscripts. Reviewers are asked to complete a checklist review form and are provided with free text boxes to elaborate on their assessment. These free text comments are reproduced below.

ARTICLE DETAILS

Title (Provisional)

Development of a resilience assessment tool for cardiac care pathways in Europe: A mixed-methods study

Authors

V. Carvalho, Ana Sofia; Brito Fernandes, Óscar; Piek, Jan J; Mauri, Josepa; Sanz Escartin, Ariadna; Wijns, William; Klazinga, Niek; Kringos, D

VERSION 1 - REVIEW

**Reviewer**	**1**
**Name**	**Volgman, Annabelle**
**Affiliation**	**Rush University Medical Center, Cardiology**
**Date**	**10-Oct-2025**
**COI**	**None**

This is a well-written and important study. Thank you for all the work put into doing this.

**Reviewer**	**2**
**Name**	**Barolia, Rubina**
**Affiliation**	**Aga Khan University**
**Date**	**20-Oct-2025**
**COI**	**None**

This is a highly interesting and relevant study. The background clearly highlights the significance of developing a resilience assessment tool to ensure continuity in cardiac care. I have provided a few minor comments that, once addressed, will help further strengthen the methodology section.

The topic is highly interesting and relevant as well, the background clearly shows the significance to have a resilience assessment tool for continuity in cardiac care, which can be affected during any new outbreak or crises; therefore, for sustainability in cardiac care the specific pathways are needed to prevent complications and deaths.

The objective is clearly mentioned in the article, but research questions have been implied under the aim, could be explicitly mentioned in a separate heading.

Methodology: The study has applied the mixed method (exploratory sequential study design) started with qualitative phase followed by quantitative that is best suitable to design/ develop a new tool. Authors have given details of all the steps followed in methodology including survey development, implementation and focus group discussions and tool development.

Results & discussions: Results have been presented and discussed logically. The quantitative data has been analyzed through descriptive and qualitative information went through thematic analysis. The data from qualitative and quantitative phase has been integrated clearly that give the credibility. Provide one or two examples.

The Findings based on the context may not be as generalizable in the context of resource-constrained settings (LMIC) so may needed to be tailored accordingly.

Reviewer 3

Name Sicari, Rosa

Affiliation National Research Council, Institute of Clinical Physiology-Pisa

Date 30-Oct-2025

COI None

This is an interesting and timing manuscript trying to understand what went wrong during Covid-19 pandemic. Authors prepared a thorough questionnaire to several EU (+ Ukraine) Cardiology departments to check how they handled cardiology emergencies during the pandemic. Moreover, after evaluating the responses they provide a toolkit to be used in case of a new pandemic in the future. The study is well conducted, the methodology is sound and the conclusions are supported by results. There are some, albeit minor, issues to be addressed.

1. Authors list in the limitations of the study low response rate to the questionnaire. This is not surprising but they probably should weigh in their statistical analysis the fact that most centers were from Italy and Spain.

2.It is clear that the lack of preparedness to such an unexpected event was a key factor in the hectic management of hospitals especially in the first weeks of the spread of the disease. It would be interesting to know which countries were better suited to face the pandemic.

3. The discussion is interesting and well written; however, one major actor who should be involved is the political decisions on these matters. The constructive parts of the study should probably be shared with the Ministry of Health of the EU countries.

4. Only GISE was involved in the study. Were other scientific societies requested to participate?

VERSION 1 - AUTHOR RESPONSE

Reviewer 1

This is a well-written and important study. Thank you for all the work put into doing this.

Response: We would like to thank Reviewer 1 for the positive comment and appreciation of our work.

Reviewer 2

This is a highly interesting and relevant study. The background clearly highlights the significance of developing a resilience assessment tool to ensure continuity in cardiac care. I have provided a few minor comments that, once addressed, will help further strengthen the methodology section.

The topic is highly interesting and relevant as well, the background clearly shows the significance to have a resilience assessment tool for continuity in cardiac care, which can be affected during any new outbreak or crises; therefore, for sustainability in cardiac care the specific pathways are needed to prevent complications and deaths.

Response: We would like to thank Reviewer 2 for the appreciation of our work and for the constructive suggestions.

The objective is clearly mentioned in the article, but research questions have been implied under the aim, could be explicitly mentioned in a separate heading.

Response: We thank the reviewer for this suggestion. We would like to mention that we have carefully followed the journal's guidelines in structuring our manuscript. The research questions are presented separately at the end of the 'Background' section. For this reason, we consider that it is not necessary to separate the research questions further, as their current placement ensures clarity and visibility for the reader.

Methodology: The study has applied the mixed method (exploratory sequential study design) started with qualitative phase followed by quantitative that is best suitable to design/ develop a new tool.

Authors have given details of all the steps followed in methodology including survey development, implementation and focus group discussions and tool development. Results & discussions: Results have been presented and discussed logically. The quantitative data has been analyzed through descriptive and qualitative information went through thematic analysis. The data from qualitative and quantitative phase has been integrated clearly that give the credibility. Provide one or two examples.

Response: Thank you for this constructive suggestion. To address the reviewer's request for more clarity on the integration of quantitative and qualitative findings, we added an explicit example in the 'Methods' section, illustrating how survey results informed the focus group discussions. This example clarifies how the sequential mixed-methods design strengthened the study.

The Methods section on “Study design and setting” now reads as follows:

> “For example, survey findings identifying workforce shortages as a key disruption were used to shape focus group discussions on strategies for staff mobilisation, which in turn helped interpret and contextualise the survey results.”

The Findings based on the context may not be as generalizable in the context of resource-constrained settings (LMIC) so may needed to be tailored accordingly.

Response: We fully agree with the reviewer’s observation regarding the generalisability of the tool. The primary focus of this study was the European context. For clarity, we added this more explicitly in the aims of the study. Recognising this, at the end of the ‘Conclusion’ section, we acknowledged that future research could test and adapt these findings to other clinical areas and geographies.

The ‘Background’ section now reads as follows:

> “This study aimed at developing a resilience assessment tool for European cardiac care pathways.”

The end of the ‘Conclusion’ section now reads as follows:

> “These results are tailored to EU countries. They are expected to be transferable to other medium- and high- income contexts, with similar health systems challenges and priorities. Future research could test and adapt these findings to other clinical areas and geographies, contributing to develop more efficient, equitable and patient-centred health systems across Europe and globally.”

Reviewer 3

This is an interesting and timing manuscript trying to understand what went wrong during Covid-19 pandemic. Authors prepared a thorough questionnaire to several EU (+ Ukraine) Cardiology departments to check how they handled cardiology emergencies during the pandemic. Moreover, after evaluating the responses they provide a toolkit to be used in case of a new pandemic in the future. The study is well conducted, the methodology is sound and the conclusions are supported by results. There are some, albeit minor, issues to be addressed.

Response: We would like to thank Reviewer 3 for the appreciation of our work and for the constructive suggestions provided.

1. Authors list in the limitations of the study low response rate to the questionnaire. This is not surprising but they probably should weigh in their statistical analysis the fact that most centers were from Italy and Spain.

Response: We thank the reviewer for this suggestion. While we acknowledge the low response rate to the questionnaire, our primary goal was not to conduct a country-weighted statistical analysis but

rather to ensure applicability of the findings across Europe. To achieve this, we complemented the survey with focus groups to validate and enrich the results through diverse stakeholder perspectives from different European countries. This mixed-methods approach ensured that the conclusions are not solely driven by the responses from Italy and Spain, but reflect broader European applicability.

This fact is acknowledged in the 'Discussion' section, as follows:

> "This study has some limitations. We obtained a low survey response rate, with an overrepresentation of Italian and Spanish cardiologists, which limits the ability to inform how each resilience dimension should be prioritised to enhance the resilience of cardiac care pathways. Nonetheless, Italy was the first European country severely impacted by the COVID-19 crisis in Europe [32] , making it, a highly relevant context from which to gather insights. Furthermore, the sequential mixed-method design allowed to discuss and complement the quantitative survey's findings in the three focus groups with stakeholders from other European countries."

2. It is clear that the lack of preparedness to such an unexpected event was a key factor in the hectic management of hospitals especially in the first weeks of the spread of the disease. It would be interesting to know which countries were better suited to face the pandemic.

Response: We thank the reviewer for this comment. Our primary objective was to learn from the challenges encountered by stakeholders during the COVID-19 pandemic and to identify key aspects that can enhance health systems' ability to cope with shocks. The aims and methodology of the study do not allow us to draw conclusions about each country preparedness. Nonetheless, strategies that proved effective were compiled and highlighted in the results, together with relevant actions that can be applied to enhance resilience to face future crises.

3. The discussion is interesting and well written; however, one major actor who should be involved is the political decisions on these matters. The constructive parts of the study should probably be shared with the Ministry of Health of the EU countries.

Response: We appreciate the reviewer's recognition of the relevance of our results and we agree that dissemination to policy-makers is essential to maximise their impact. As such, the RESIL-Card Consortium is actively engaged, through dedicated work streams, in dialogue with both national and European policy-makers. Various initiatives are underway to connect with Ministries of Health to share the findings of this study more broadly. We included a reference to this aspect in the "Discussion" section, which reads as follows:

> "The RESIL-Card Consortium is actively engaged, through dedicated work streams, in disseminating the RESIL-Card tool to promote its broader adoption across Europe, and encouraging patient involvement during its use. Ongoing activities include dialogue with national and European policy makers, scientific societies and care providers."

4. Only GISE was involved in the study. Were other scientific societies requested to participate?

Response: We thank the reviewer for this comment. We would like to clarify that this project was submitted and co-funded by the European Union on behalf of 4 Consortium members: We Care, AMC, Catsalut and GISE. Indeed, as one of the Consortium members, GISE was actively involved in engaging with stakeholders and in reviewing the tool, as described in the study. GISE will also be in charge of the dissemination of the tool, primarily in Italy.

Nonetheless, as described in the 'Methods' section, the medical community has been invited to participate from the onset of this study. Specifically, through sharing of experience, survey participation, and involvement in focus groups. Through We Care, the Consortium has access to a large community of health care professionals in the field and many stakeholders will also be invited to engage at the stage of dissemination (starting end of Q1 2026). These include all European members of the network of national working groups and interventional cardiology societies.

The dissemination of the survey to scientific communities is explained in the 'Methods' section, as follows:

> "The data collection comprised a period of six weeks, between 5th May and 16th June 2024. A link to the questionnaire was sent via email to 4338 European members of 'PCR companions' community, an international network of interventional cardiologists and allied professionals, followed by one reminder and reference to the survey in two additional communications. Various partner organisations contributed to the dissemination of the questionnaire, including Societies of Cardiology from the targeted countries."

Furthermore, various scientific societies were involved in the focus groups. Some examples are: Spanish Society of Cardiology, Italian Federation of Healthcare and Hospital Companies, Emergency Medical Services (EMS) Europe, *Ambulancezorg Nederland* (National sector organization for ambulance care in The Netherlands), Dutch Society of Cardiology (*Nederlandse Vereniging voor Cardiologie*; NVVC). In the study, these were initially listed as "*professional associations*" in Table 1. To enhance accuracy, we have revised this stakeholder type to "*scientific society*" in Table 1.
